# The Effects of Ivermectin on *Brugia malayi* Females *In Vitro*: A Transcriptomic Approach

**DOI:** 10.1371/journal.pntd.0004929

**Published:** 2016-08-16

**Authors:** Cristina Ballesteros, Lucienne Tritten, Maeghan O’Neill, Erica Burkman, Weam I. Zaky, Jianguo Xia, Andrew Moorhead, Steven A. Williams, Timothy G. Geary

**Affiliations:** 1 Institute of Parasitology, Centre for Host-Parasite Interactions, McGill University, Sainte-Anne-de-Bellevue, Quebec, Canada; 2 Department of Infectious Diseases, College of Veterinary Medicine, University of Georgia, Athens, Georgia, United States of America; 3 Filariasis Research Reagent Resource Center, Smith College, Northampton, Massachusetts, United States of America; 4 Department of Biological Sciences, Smith College, Northampton, Massachusetts, United States of America; Yale School of Public Health, UNITED STATES

## Abstract

**Background:**

Lymphatic filariasis and onchocerciasis are disabling and disfiguring neglected tropical diseases of major importance in developing countries. Ivermectin is the drug of choice for mass drug administration programs for the control of onchocerciasis and lymphatic filariasis in areas where the diseases are co-endemic. Although ivermectin paralyzes somatic and pharyngeal muscles in many nematodes, these actions are poorly characterized in adult filariae. We hypothesize that paralysis of pharyngeal pumping by ivermectin in filariae could result in deprivation of essential nutrients, especially iron, inducing a wide range of responses evidenced by altered gene expression, changes in metabolic pathways, and altered developmental states in embryos. Previous studies have shown that ivermectin treatment significantly reduces microfilariae release from females within four days of exposure *in vivo*, while not markedly affecting adult worms. However, the mechanisms responsible for reduced production of microfilariae are poorly understood.

**Methodology/Principal Findings:**

We analyzed transcriptomic profiles from *Brugia malayi* adult females, an important model for other filariae, using RNAseq technology after exposure in culture to ivermectin at various concentrations (100 nM, 300 nM and 1 μM) and time points (24, 48, 72 h, and 5 days). Our analysis revealed drug-related changes in expression of genes involved in meiosis, as well as oxidative phosphorylation, which were significantly down-regulated as early as 24 h post-exposure. RNA interference phenotypes of the orthologs of these down-regulated genes in *C*. *elegans* include “maternal sterile”, “embryonic lethal”, “larval arrest”, “larval lethal” and “sick”.

**Conclusion/Significance:**

These changes provide insight into the mechanisms involved in ivermectin-induced reduction in microfilaria output and impaired fertility, embryogenesis, and larval development.

## Author Summary

Lymphatic filariasis and onchocerciasis are tropical diseases caused by infections with parasitic nematodes. Resulting chronic diseases can be strongly blinding and disfiguring, and contribute to an entrenched cycle of poverty in affected populations. Ivermectin is one of the pivotal drugs used to control these infections. The mechanism of antifilarial action of the drug is incompletely resolved. It kills circulating larval stages (microfilariae), but only reversibly sterilizes adult worms without killing them. Our limited understanding of the involved mechanisms hampers treatment optimization and sustainability of the efficacy of the drug, and investigations into its pharmacology are of paramount importance. Working with *Brugia malayi* adult females, we employed RNA sequencing and bioinformatics analyses to identify genes for which expression levels changed as a result of exposure to the drug *in vitro*. Ivermectin exposure altered the expression of genes that are likely to function in the *B*. *malayi* female reproductive system even at the lowest concentration tested. Through several biological pathways, genes involved in meiosis were particularly affected. These findings provide some insight into the mechanisms involved in ivermectin-induced reduction in microfilaria output and impaired fertility, embryogenesis, and larval development.

## Introduction

Lymphatic filariasis (LF) is caused by the transmission of *Wuchereria bancrofti*, *Brugia malayi*, and *Brugia timori* by infected mosquitos and is endemic in 60 countries, mainly in subtropical and tropical regions, with over 120 million people infected [1]. Onchocerciasis (river blindness) is caused by *Onchocerca volvulus* and afflicts 25 million people in 24 countries, mainly in Sub-Saharan Africa [2]. Long-running control and elimination programs aim to reduce transmission of both infections by mass drug administration (MDA) of microfilaricidal drugs and to alleviate suffering and disability in onchocerciasis patients [3, 4].

The macrocyclic lactones (ML) are a class of broad-spectrum anthelmintic drugs which include the avermectins and milbemycins (e.g., ivermectin (IVM) and moxidectin, respectively) [5]. Although similar in structure, they differ in pharmacokinetics and dynamics as well as physicochemical properties [6, 7]. These drugs have been extensively used in veterinary medicine for the treatment or prevention of nematode infections and ectoparasite infestations. In human medicine, IVM is one of the three mainstay drugs in the LF global elimination program [8] and is the only drug used in onchocerciasis MDA campaigns [2]. IVM is predominantly microfilaricidal, the killing effect on adults being only moderate and requiring repeated treatment cycles over several years [9].

IVM pseudo-irreversibly activates nematode and arthropod glutamate-gated chloride channels (GluCls), resulting in hyperpolarization of neurons and pharyngeal muscle cells, leading to paralysis of movement and pharyngeal pumping, and ultimately starvation of the worm [10–12]. Treatment results in a dramatic reduction in microfilaria (mf; first-stage larvae (L1) production by adult female filariae, but the mechanisms involved are not understood. GluCl expression in *B*. *malayi* mf was localized to a muscle structure surrounding the excretory-secretory (ES) vesicle, suggesting that release of proteins was regulated by GluCls (14). In the same study, IVM exposure resulted in a significant reduction of protein release, possibly preventing the secretion of proteins that allow evasion of the host’s immune system [13]. Recently, the *B*. *malayi* GluCl gene *avr*-14 was found to be highly expressed in reproductive tissues and embryos, suggesting an involvement of GluCls in gamete production and embryogenesis which may account for the sterilizing effect observed in adult filarial worms treated with IVM [14]. Our limited understanding of IVM’s mode of action on adult filarial worms hampers treatment optimization and sustainability of the efficacy of this drug, and investigations into its pharmacology are of paramount importance.

We used adult female *B*. *malayi*, a well-characterized model of filarial infections, to study the time-dependent transcriptomic changes induced by exposure to IVM *in vitro*. We performed two separate studies: 100 nM IVM exposure for up to 72 h, and 300 nM and 1 μM for up to 5 days, to gain insight into the mechanisms which underlie the drug-induced long-term sterility of adult females and subsequent reduction of mf in skin or blood of treated patients. The drug concentrations used in this present study have previously been tested on adult *B*. *malayi* females *in vitro* [15] and have also been tested in *C*. *elegans* [7]. The rationale for using the lowest concentration (100 nM) in the first study was to expose worms to a concentration similar to plasma levels detected in humans after administration of a dose typically used in MDA [16–18]. It is recommended that *in vitro* experimental analyses of drug effects include concentrations higher than the maximal blood/plasma concentration achieved in an animal [19]. We therefore tested higher IVM concentrations in a second phase. These results allowed us to also compare our results with those previously observed in *B*. *malayi* at these concentrations.

## Materials and Methods

### Ethics statement

All animal procedures were approved by the University of Georgia, Institutional Animal Care and Use Committee, and complied with U.S. Department of Agriculture's regulations (USDA Assurance No. A3437-01)

### Parasites and study designs

Adult male jirds (*Meriones unguiculatus*) were infected subcutaneously with ≈400 infective *B*. *malayi* larvae (L_3_). After a minimum of 90 days post-infection (ranging from 3 to 6 months), jirds were euthanized by exposure to CO_2_ and adult worms were collected from the peritoneal cavity via lavage.

### Study design

#### First study

**Fig 1:**
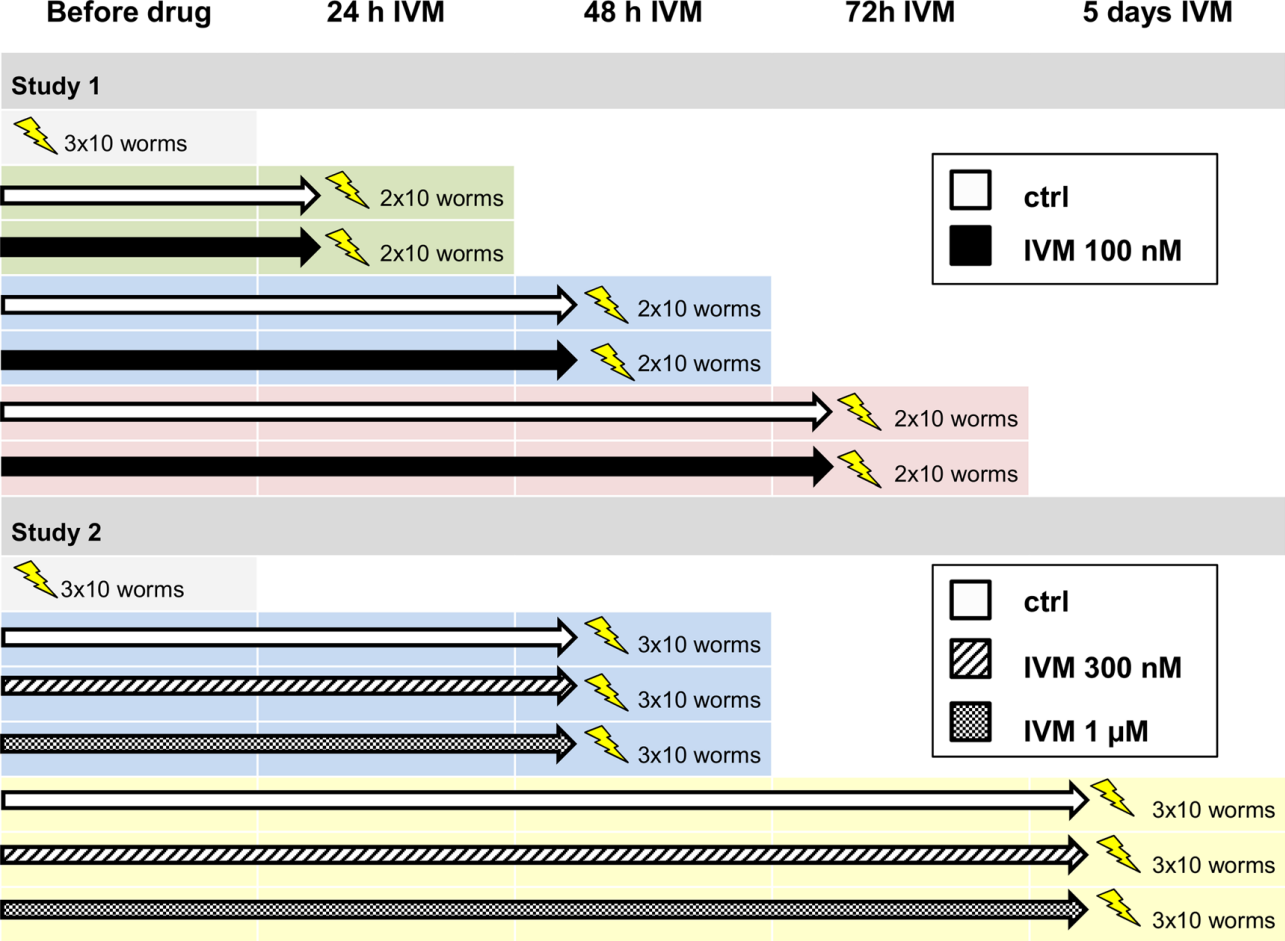
Study design. At each time point, two or three groups of 10 worms were washed, flash-frozen (represented by lightning bolt) and used for RNA extraction. IVM: ivermectin.

Female worms from three individual jird hosts were randomly assigned to 15 groups (9 untreated and 6 exposed to 100 nM IVM) of 10 worms each. The study design consisted of 4 time points (before drug exposure, 24, 48, and 72 h). Three technical replicates were used before drug exposure and duplicates for the remaining conditions (Fig 1).

#### Second study

Female worms from three individual jird hosts were randomly assigned to 21 groups (9 untreated, 6 exposed to 300 nM IVM, and 6 exposed to 1 μM IVM) of 10 worms each. The study design consisted of 3 time points (before drug exposure, 48 h and 5 d). Three technical replicates were used for each condition (Fig 1).

For both studies, worms were collected from jirds at the NIH-NIAID Filariasis Research Reagent Resource Center (FR3) at the University of Georgia and separated by sex. Worms were then shipped overnight in separate tubes of 15 ml culture media containing RPMI-1640 (BioWhittaker Classic Cell Culture Media, Lonza Walkersville Inc.) and 1% v/v gentamycin (gentamycin solution, 10 mg/ml Sigma Aldrich Inc.) to McGill University for further analysis. The separate studies were conducted several months apart.

Upon arrival at McGill, the first group (before drug exposure) was thoroughly washed several times in PBS and flash-frozen in liquid N_2_ for RNA extraction.

Remaining worms were incubated 1 worm per well of a 6-well plate (Costar) containing 6 ml RPMI 1640 (Sigma-Aldrich) supplemented with 10% v/v heat-inactivated fetal bovine serum (Sigma-Aldrich, F1051), 5% penicillin/streptomycin (Sigma–Aldrich P4333) and 2% v/v gentamycin (Gibco, 15750–060). Worms were cultured for another 24, 48, or 72 h (first study) and 48 h or 5 days (second study) at 37°C, 5% CO_2_ with or without exposure to IVM (Sigma-Aldrich I8898) at 100 nM (study 1), 300 nM or 1 μM (study 2). Control wells contained 0.1% v/v DMSO. On a daily basis, 3 ml of culture media (with or without drug at the different concentrations) in each well was replaced with fresh media.

### RNA extraction

RNA extraction was performed as described previously [20]. Briefly, live worms were washed several times with PBS; immotile worms were excluded from the study. RNA was extracted from 5 to 8 worms per group using nuclease-free reagents. On ice, 125 μl 0.1X Tris-EDTA (TE) buffer, pH 8.0 (Ambion, Life Technologies, Burlington, ON) and 375 μl Trizol LS reagent (Ambion) were added to each tube. Worm extracts were obtained by conducting three cycles of flash-freezing in liquid N_2_ followed by crushing of worms using plastic pestles. One-hundred (100) μl chloroform was added to each sample in a 1.5 ml Eppendorf tube, which was vortexed and incubated 3 min at room temperature. The extracts were transferred to phase-lock gel heavy tubes (5 PRIME, Gaithersburg, MD) and centrifuged at 11,900 x *g* at 4°C for 15 min for phase separation. The aqueous phase (approximately 150 μl) was transferred to fresh 1.5 ml Eppendorf tubes and mixed with 250 μl ice-cold isopropanol. RNA was precipitated by centrifugation at 12,200 x *g* at 4°C for 30 min followed by an overnight incubation at -20°C. Pellets were washed twice with 80% EtOH, allowed to dry and resuspended in 50 μl 0.1X TE prior to heating at 55°C for 10 min. Total RNA was purified and concentrated on columns (RNeasy Min-Elute Cleanup Kit, Qiagen, Valencia, CA). Samples were treated with DNase (Ambion DNA-free Kit, Life Technologies, AM1906, Burlington, ON) according to the manufacturer’s instructions to remove contaminating DNA. Assessment of RNA concentration and purity was done by spectrophotometry (NanoDrop 1000, Wilmington, DE) and RNA samples were shipped overnight on dry ice to the NIH-FR3 (Molecular Division) at Smith College (Northampton, MA) for cDNA library preparation and Illumina sequencing. RNA concentration, purity and integrity were precisely measured using the Qubit RNA BR Assay Kit (Life Technologies, Q10210, Burlington, ON) and on an Agilent 2100 Bioanalyzer (Santa Clara, CA).

### cDNA library preparation and Illumina sequencing

Messenger RNA was isolated with a NEBNext Poly (A) mRNA Magnetic Isolation Module (New England Biolabs, E7490). The enriched mRNA served as template for cDNA library preparation with a NEBNext Ultra RNA Library Prep Kit Illumina (NEB, E7530) and NEBNext Multiplex Oligos for Illumina (Index Primer 1–12) (NEB, E7600) following the manufacturer’s instructions. Assessment of quality, concentration and size of the cDNA, was performed for each library using a Qubit 2.0 Fluorometer (Life Technologies, Q32866), Qubit dsDNA BR assay kit (Life Technologies, Q32850), High Sensitivity DNA Analysis Kit (Agilent, 5067–4626) and Agilent 2100 Bioanalyzer. cDNA libraries were sequenced on an Illumina MiSeq Platform employing a 150 base pair paired-end NGS setting. Sequencing data were uploaded and stored in BaseSpace (https://basespace.illumina.com) for subsequent analysis.

## Data analysis

### RNA sequencing analysis

Procedures for data analysis were previously described [20]. Unaligned raw sequencing data (fastq) files were downloaded to the Mason-Galaxy platform hosted at Indiana University (http://galaxy.iu.edu/) [21, 22]. Raw sequence data were first processed using Fastq Groomer (v 1.0.4) to convert file format to Fastq Sanger format and quality scores were verified using FastQC (version 0.52). Based on quality statistics, sequences were trimmed from both the 5’ and 3’ ends using Fastq Quality Trimmer (v 1.0.0) and Trim Galore (v 0.2.8.1). Mapping of gapped reads to the *B*. *malayi* reference genome (ftp://ftp.wormbase.org/pub/wormbase/species/b_malayi/sequence/genomic/b_malayi.PRJNA10729.WS243.genomic.fa.gz) was performed with Tophat2 (v 0.6) and RNA sequencing metrics were retrieved using Picard tools (Table 1). Picard alignment summary metrics (http://broadinstitute.github.io/picard/) were generated from BAM files using SAM/BAM Alignment Summary Metrics (version 1.56.0) tool. The read count table was then generated by counting the number of reads per gene feature with HTSeq-count (version 1.0.0) [23] using the union mode to handle reads covering more than one feature and default settings for all other parameters. Differential gene expression analysis between time points and drug concentrations was realized in edgeR (version 3.10.5) [24] through the web interface provided by NetworkAnalyst (http://networkanalyst.ca) [25, 26]. To correct for different library sizes and reduce RNA compositional effects, read counts for gene features were normalized in edgeR using the trimmed mean of M-values (TMM) method [27]. The empirical Bayes method based on weighted conditional maximum likelihood was employed to estimate tagwise (gene-specific) dispersion values [28]. Once negative binomial models were fitted and dispersion values estimated, the exact test was used for pairwise differential expression testing between treated and untreated groups. Significance was set as an experiment-wide false discovery rate (FDR) <0.20 (after the Benjamini-Hochberg method [29]). After filtering, gene lists for each pairwise comparison were imported into Microsoft Excel for further analysis.

**Table 1:** Picard alignment summary metrics. Summary of the sequencing and mapping of the data to the *B*. *malayi* transcriptome.

	Time point	Concentration	Total # Transcripts	Ave Total # Reads	Ave Total # Reads Mapped	Ave Total # High Quality Reads Mapped	% High Quality Reads Mapped
**Study 1**	**24 h**	IVM (100 nM)	10691	3537601	3537500	2990362	84.53
		Control	10511	2765626	2765551	2364245	85.49
	**48 h**	IVM (100 nM)	10086	2513891	2513834	2145661	85.35
		Control	10214	2957918	2957831	2519799	85.19
	**72 h**	IVM (100 nM)	10705	3875640	3874592	3380930	87.24
		Control	10428	3081545	3081499	2683543	87.09
**Study 2**	**48 h**	IVM (300 nM)	10087	2091222	2091198	1710598	81.80
		IVM (1 μM)	10177	2212133	2212085	1757015	79.43
		Control	10328	2808908	2808880	2325764	82.80
	**5 d**	IVM (300 nM)	9959	1799180	1799161	1456538	80.96
		IVM (1 μM)	9837	1798183	1798159	1426230	79.32
		Control	10250	2464701	2464671	2017612	81.86

Common dispersion, which provides information on the overall variability across the genome for a dataset, and Biological Coefficients of Variation (BCV) were calculated; BCV plots were generated using the edgeR Bioconductor package (version 3.12.0) [24].

#### Bioinformatics analysis of the sequence data

From lists of retained genes for each comparison, the Wormbase Gene ID was used to retrieve the primary corresponding sequence ID of the gene from Wormbase (http://www.wormbase.org/) and the UniProt accession number [30]. Protein coding sequences from Wormbase were used in BLASTn in order to find the closest *B*. *malayi* gene hit and attribute a GenBank ID and annotation to each gene. Gene Ontology (GO) terms [31] were obtained when available from nematode.net (v 4.0; http://nematode.net/NN3_frontpage.cgi) [32, 33]. Enriched GO terms and KEGG/Panther pathways were retrieved and analyzed using WebGestalt (WEB-based Gene SeT AnaLysis Toolkit) [34]. Pathways and GO terms were considered significantly enriched when FDR<0.05. Since many DE genes detected in this study were uncharacterized or not annotated yet had orthologs which could be identified in the *Caenorhabditis elegans* genome, we used the Uniprot accession numbers of the *C*. *elegans* orthologs of *B*. *malayi* differentially expressed (DE) genes for enrichment analysis, accepting hits with a minimal E-value of 1*10^−20^ and using the Blast-based Reciprocal Best Hit (RBH) strategy for identifying orthologs [35, 36]. RNA interference (RNAi) phenotypes associated with the *C*. *elegans* orthologs of down-regulated genes at 48 h with 100 nM IVM exposure involved in reproduction, embryo and larval development were retrieved from www.wormbase.org. A hypergeometric test was performed to determine which phenotypes in this dataset were significantly enriched when compared with their overall occurrence in the *C*. *elegans* RNAi dataset. P-values were corrected for multiple testing using the Benjamini-Hochberg method (1995) with a level of significance of q<0.05.

## Results

### Sequencing and mapping

Table 1 displays the total number of transcripts obtained for each sample in both studies and the number of sequence reads for each cDNA library. On average for study 1 (100 nM IVM), 85.82% of the high quality sequence reads were mapped to the *B*. *malayi* transcriptome after removal of ambiguous sequence matches (Table 1). Gene mapping fluctuated from 1 to 58487 sequence read(s) per gene. On average for study 2 (300 nM and 1 μM IVM), 81.03% of the high quality sequence reads were mapped to the *B*. *malayi* transcriptome after removal of ambiguous sequence matches, and gene mapping fluctuated from 1 to 27507 sequence read(s) per gene (Table 1). In study 1, 93.28–95.79% of reads aligned in pairs to a transcript; in study 2, 90.43–93.52% of reads aligned in pairs.

BCV values ranged from 17 to 27% in the first study and 15 to 35% in the second study (S3 Table). BCV plots show that the BCV values in the first study tended to increase over time and are shown in S1A–S1C Fig. In the second study, the BCV values were higher at the 48 h time point than at 5 days for both IVM concentrations.

We used gene expression levels from untreated (control) worms at each time point as a baseline to identify genes with differential expression after exposure to IVM in each sample. Pairwise comparisons revealed 15 (100 nM, 72 h) to 421 (100 nM, 48 h) DE genes (Table 2, S1 Table). Between 20.59 and 65.15% of DE genes could be assigned GO terms, while similar proportions had a *C*. *elegans* ortholog.

**Table 2:** Numbers of differentially expressed genes over time after IVM exposure in culture.

	100 nM IVM			300 nM IVM		1 μM IVM	
	24 h	48 h	72 h	48 h	5 d	48 h	5 d
Total DE genes	34	421	15	132	68	147	271
Total DE genes up-regulated	21	141	2	14	24	136	74
Total DE genes down-regulated	13	280	13	118	44	11	197
Sequences with GO terms*	7	178	9	86	29	49	160
Sequences with *C*. *elegans* ortholog	7	257	9	92	37	64	186

*GO terms were provided by Nematode.net V.4 [33]. IVM: ivermectin; DE: differentially expressed

### Transcriptomic profiles of worms exposed to IVM

The greatest overlap of DE genes was seen between the 300 nM and 1 μM groups, with 66 dysregulated genes. When comparing the overlap of DE genes from the first and second study, only 5 genes were found to overlap across the 3 concentrations, with 10 genes in common between the 100 nM and 300 nM groups and 20 genes in common between the 100 nM and 1 μM groups. GO term enrichment showed that 5 of these genes were enriched in “structural molecule activity” molecular function (GO:0005198; FDR = 0.009). Five genes were common to all three IVM treatments (Bm8439 and Bm4605 (two collagens), Bm9776, Bm6450, and Bm9380). Bm9380 is a serpin precursor (Bm-spn-2) and was significantly up-regulated in both studies (100 nM 72 h; 300 nM and 1 μM 5 days; log_2_ fold-changes 1.37–2.57). Bm9776 encodes a hypothetical protein and was also significantly up-regulated at four time points between both studies (log_2_ fold-changes 1.06–1.85). Lastly, Bm6450 is annotated as a GTPase activating protein for Arf containing protein and was found to be down-regulated across three different time points between both studies (log_2_ fold-changes -1.06 to -1.43).

#### Study 1: Transcriptomic profiles of worms exposed to 100 nM IVM

At 100 nM IVM, 460 DE genes were identified across all time points. Worms exposed to 100 nM IVM for up to 72 h showed the largest change in their transcriptome profiles at 48 h post-exposure (421 DE genes), at which time 67% of those genes were down-regulated (S1 Table, Table 2). Among them, a dynein light chain type 1 family protein, Bm8648, was down-regulated (log_2_ fold-change: -1.46), as well as Bm14080 (log_2_ fold-change: -1.51). A cytidine deaminase (Bm4230) was the gene most down-regulated at the 48 h and 72 h time points (log_2_ fold-change: -5.21 and -6.90, respectively), while the most prominently up-regulated gene in study 1 was Bm8966 (hypothetical protein; log_2_ fold-change: 6.91; 72 h 100 nM). To cite only a few interesting dysregulated genes at 48 h or 72 h, *Bma-nep-1*, a neprilysin (Bm7419, log_2_ fold-change: -3.92), 6 enzymes involved in oxidative phosphorylation (Bm6047, Bm3783, Bm3758, Bm5403, Bm10539, Bm2497; log_2_ fold-changes between -0.85 and -1.53), and inositol-1 (Bm11145; log_2_ fold-change: -4.48) were among down-regulated genes. Eight DE genes overlapped across the 24 h and 48 h time points (among them, a kinesin light chain protein 2 (Bm9032), up-regulated at 24 h (log_2_ fold-change: 2.10) but then significantly down-regulated by 72 h (-3.23 log_2_ fold-change); only one common DE gene was found at both 48 h and 72 h. One DE gene was shared between 24 h and 72 h (but not with 48 h).

Exposure to 100 nM also resulted in the significant down-regulation (-1.16 to -2.83 log_2_ fold) of five cuticle collagens at all time points.

**Fig 2:**
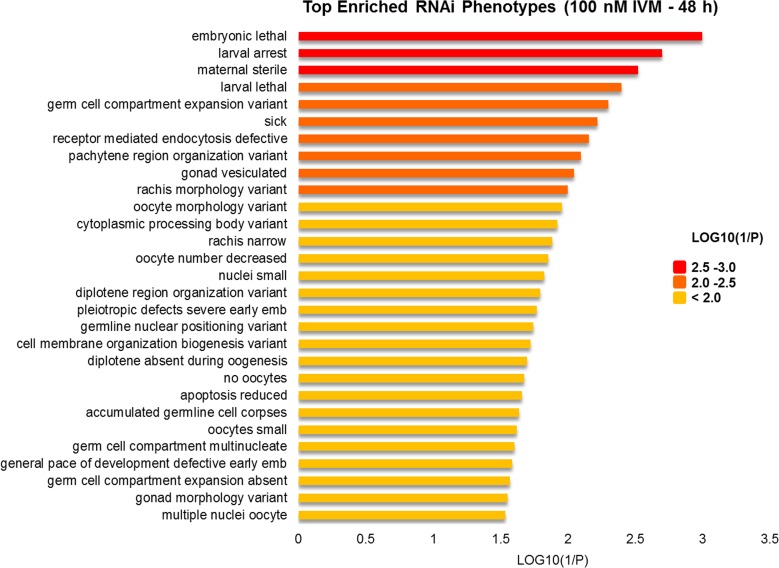
Top RNAi phenotypes enriched in reproductive processes after 48 h exposure to 100 nM IVM. The listed enriched phenotypes obtained were based on Gene Ontology analysis of *C*. *elegans* orthologs of DE genes. These phenotypes were significantly overrepresented compared with their overall abundance in the *C*. *elegans* RNAi phenotype dataset in Wormbase. Statistical method: Hypergeometric; *p*-value adjusted using the Benjamini-Hochberg multiple test adjustment (FDR<0.05).

Gene Ontology enrichment analysis of the 100 nM (48 h) dataset revealed significant enrichment of genes with GO terms associated with “growth” (GO:0040007; FDR = 2.03e-08), “locomotion” (GO:0040011; FDR = 2.99e-07) and developmental processes which included “embryo development” (GO:0009790; FDR = 1.07e-06), “embryo development ending in birth or egg hatching” (GO:0009792; FDR = 1.07e-06) and “nematode larval development” (GO:0002119; FDR = 1.07e-06), with 63–91 genes enriched in these categories. Table 3 shows the distribution of the most enriched GO terms at 100 nM IVM exposure for 48 h, for which reproductive processes (reproduction, embryo development and larval development) were statistically overrepresented in the dataset. RNAi phenotypes which were significantly overrepresented in the down-regulated genes dataset compared with their overall abundance in the *C*. *elegans* RNAi phenotype dataset in Wormbase are shown in Fig 2 and S4 Table (FDR < 0.05; hypergeometric test). The most commonly observed RNAi phenotype is “embryonic lethal”. Over 81% of down-regulated genes involved in reproduction were associated with an embryonic lethal RNAi phenotype, while 62% were linked to a larval arrest phenotype. At the 24 h time point, no significantly enriched biological processes or molecular functions were noted. “Cytoskeleton” was the greatest enriched cell component term (GO:0005856; FDR = 0.007).

**Table 3:** Gene Ontology enrichment analysis of top 15 biological processes at 100 nM IVM exposure for 48 h.

Gene Ontology Term*	GO Identity	Observed	Expected	Reference	Ratio of Enrichment	FDR value**
growth	GO :0040007	85	45.16	2498	1.88	2.03^E^-08
locomotion	GO :0040011	63	30.10	1665	2.09	2.99^E^-07
embryo development	GO :0009790	91	55.49	3069	1.64	1.07^E^-06
embryo development ending in birth or egg hatching	GO :0009792	89	54.10	2992	1.65	1.07^E^-06
nematode larval development	GO :0002119	72	39.00	2157	1.85	1.07^E^-06
larval development	GO :0002164	72	39.03	2159	1.84	1.07^E^-06
post-embryonic development	GO :0009791	72	39.31	2174	1.83	1.07^E^-06
establishment of localization	GO :0051234	65	33.63	1860	1.93	1.07^E^-06
transport	GO :0006810	63	32.33	1788	1.95	1.21^E^-06
regulation of growth	GO :0040008	61	31.24	1728	1.95	1.96^E^-06
positive regulation of growth	GO :0045927	59	29.90	1654	1.97	2.32^E^-06
positive regulation of growth rate	GO :0040010	54	26.27	1453	2.06	2.38^E^-06
regulation of growth rate	GO :0040009	54	26.31	1455	2.05	2.38^E^-06
cellular localization	GO :0051641	26	7.85	434	3.31	2.38^E^-06
reproduction	GO :0000003	80	48.00	2655	1.67	3.76^E^-06

*Analysis performed using Web-Based Gene Set Analysis Toolkit (WebGestalt).

**Statistical method: Hypergeometric; *p*-value adjusted using the Benjamini-Hochberg multiple test adjustment.

#### Study 2: Exposure to higher IVM concentrations

In the second study, most changes occurred after 48 h exposure to 300 nM IVM, and only after 5 days when exposed to 1 μM. The expression of 535 genes was dysregulated across several time points and/or concentrations (S1 Table). As the BCVs varied with time and across concentrations, resulting in higher FDRs (as BCVs increased), it was difficult to see a concentration-dependent trend across the DE genes detected in the study and therefore the overlap between DE genes across concentrations and time points was very limited.

At 48 h, only 3 genes overlapped between the groups exposed to 300 nM and 1 μM IVM; at 5 days, 18 genes overlapped between both treatment groups. Among them, 3 genes encoding cuticle collagens (Bm8043, Bm4605, and Bm11024; log_2_ fold-changes -0.84–-2.23), and others, to cite a few only, coding for a major facilitator superfamily protein (Bm6076; log_2_ fold-changes -1.1 and -2.21), *Bma-zyg-9* (Bm3504; log_2_ fold-change -1.51 at 48 h and -1.20 at 5 days), which may play a role in meiosis, were down-regulated. Upregulated across concentrations at 5 days was a serpin precursor (Bm9380; log_2_ fold-changes 1.37 and 2.08).

#### Transcriptomic profiles of worms exposed to 300 nM IVM

Worms incubated at this concentration showed the largest change after 48 h exposure (132 DE genes). 89% of DE genes were down-regulated at this time. The most strongly down-regulated gene encodes a prominin protein (Bm3187; log_2_ fold-change: -3.84) predicted to be a 5-transmembrane domain glycoprotein. Other annotated genes of potential interest that were highly down-regulated include IQ calmodulin-binding motif family protein (Bm2777; log_2_ fold-change: -2.01), Fanconi anemia complementation group D2 protein (Bm6522; log_2_ fold-change: -2.0), CG7507-PA (Bm5897; log_2_ fold-change: -1.20), an ortholog of dynein heavy chain-1 in *C*. *elegans*, and nucleoporins (Bm6397 and Bm5288; log_2_ fold-change: -1.19 and -1.91, respectively). The most up-regulated gene was Bm12387 (log_2_ fold-change: 3.83), annotated as a hypothetical protein. Gene Ontology analysis at this time point showed an enrichment of genes related to “establishment of meiotic spindle orientation” (GO:0051296; FDR = 0.04), “meiotic spindle organization” (GO:0000212; FDR = 0.04) and “establishment of meiotic spindle localization” (GO:0051295; FDR = 0.04) (S2A Table).

By 5 days, the number of DE genes decreased from 132 to 68. Bm11304, a KH domain containing protein, was the most prominently down-regulated gene at this time point and concentration (log_2_ fold-change: -5.9), while Bm1543, a lectin C-type domain containing protein, was the most up-regulated (log_2_ fold-change: 5.88). GO enrichment analysis revealed enrichment of genes in molecular processes related to “fatty-acyl-CoA binding” (GO:0000062; FDR = 0.0057), “structural constituent of cuticle” (GO:0042302; FDR = 0.0002), and “structural molecule activity” (GO:0005198; FDR = 0.016) (S2C Table).

#### Transcriptomic profiles of worms exposed to 1 μM IVM

Exposure to 1 μM IVM resulted in the dysregulation of 147 genes at 48 h and 271 after 5 days. Most strongly down-regulated at 48 h was Bm2880, a tachykinin-like peptide receptor protein (log_2_ fold-change: -5.51), and a GRIP-domain containing protein, at 5 days (log_2_ fold-change: -5.71). At both time points, two hypothetical proteins were maximally up-regulated (log_2_ fold-change: 6.76 and 6.13, respectively). Transcriptomic data from worms exposed to 1 μM IVM for 48 h showed “translation” to be the most significantly enriched GO term (GO:0006412; FDR = 8.87e-06) (S2B Table). After 5 days exposure, down-regulation of a phospholipase C homolog (Bm3716; log_2_ fold-change: -1.54), and of 1-phosphatidylinositol-4,5-bisphosphate phosphodiesterase beta 4 (Bm1768; log_2_ fold-change: -0.89) was observed. Significantly enriched GO terms also included terms related to “double-strand break repair via nonhomologous end joining” (GO:0006303; FDR = 0.02), “cytokinesis” (GO:0007109; FDR = 0.02), “cytokinetic cell separation” (GO:0000920; FDR = 0.02), “cytokinetic process” (GO:0032506; FDR = 0.003), “cytokinesis after meiosis” (GO:0033206; FDR = 0.01) and “polar body extrusion after meiotic divisions” (GO:0040038; FDR = 0.004). Nine genes were also found enriched in the “collagen and cuticulin-based cuticle development” (GO:0040002; FDR = 0.003) category (S2D Table).

## Discussion

We performed time-series studies to assess the effects of exposure to different concentrations of IVM on adult *B*. *malayi* females *in vitro* at the transcriptomic level. Of particular interest was the identification of DE genes involved in reproduction, which could help explain the sterilizing effect of IVM on adult filariae.

Overall BCVs were calculated between samples in both drug studies and were 17–27% in the first study and 15–35% in the second study (S3 Table, S1 Fig), suggesting acceptable biological variability among replicates. Typically, BCVs for datasets from well-controlled studies range from 10% (genetically identical model organisms) to 40% (human data). In a previous study, we reported BCVs to be 14–25% for samples of adult female worms immediately upon extraction from jirds [20]. However, BCVs were slightly higher at the 48 h time point in study 2 (30 and 35%). As BCVs varied across the different concentrations and time points and resulted in higher FDRs as the BCVs increased, we were unable to see obvious trends in concentration- dependence and detected few DE genes that overlapped across the different comparisons. Therefore, our discussion is mainly centered on the analysis of the DE genes detected for each pairwise comparison. We focus on a few processes particularly affected by exposure to IVM *in vitro*.

IVM significantly reduces the release of mf after 24 h exposure to IVM *in vitro* and by 4 days, mf output ceases completely at concentrations ≥ 0.15 μg/ml [15]. In the present work, we exposed adult females at a concentration of 100 nM (0.09 μg/ml) for up to 72 h in the first study and 300 nM (0.267 μg/ml) and 1 μM (0.89 μg/ml) for up to 5 days in the second study. Although we did not measure mf output, we suspect that adult females were sterilized at these concentrations based on the previous work. In addition, *wsp* (*Wolbachia* surface protein) expression has been shown to decrease significantly with IVM concentrations >0.31 μg/ml (348 nM) [15]. It is therefore possible that some of the observed changes in gene expression might be related to changes in *Wolbachia* densities; however, we are unable to confirm this as *wsp* expression was not measured. *Wolbachia*-related death at these concentrations could have negative implications on reproduction, molting and survival. Interestingly, the *Wolbachia* surface protein wBm0432 has been shown to interact with several key enzymes involved in the host glycolytic pathway, including aldolase and enolase, and wBm0152 interacts with the host cytoskeletal proteins actin and tubulin [37]. These interactions could have an effect on growth and embryogenesis, effects we observed at the transcriptome level.

### IVM dysregulates genes involved in meiosis and calcium signaling surrounding fertilization

Dyneins and kinesins play important roles in mitosis and meiosis [38–40] and both were differentially expressed by exposure to IVM. CG7507-PA (Bm5897), orthologous to dynein heavy chain-1 in *C*. *elegans*, was down-regulated after IVM exposure. These molecular motors use ATP to move along microtubules and function in carrying cargo such as vesicles and organelles which are too large to diffuse to other cellular destinations [41]. In *C*. *elegans*, dynein is associated with nuclear envelopes, centrosomes and meiotic and mitotic spindle poles [42]. Kinesin-1 and cytoplasmic dynein function together to move the meiotic spindle to the oocyte cortex [39]. During anaphase, the meiotic spindle is attached to the cortex by one pole, allowing selective disposal of half the chromosomes in a polar body [39]. In *C*. *elegans*, dynein light chain 1 (*dylt-1*) is expressed in adult body wall muscle, larval and adult pharynx, posterior intestine and nervous system and is required for normal embryonic and larval viability. The GO term “establishment of meiotic spindle orientation” was enriched in this study. One gene associated with this term was Bm2777 (IQ calmodulin-binding motif family protein), a homolog of *aspm-1* in *C*. *elegans* and mammalian ASPM (Abnormal Spindles & Primary Microcephaly) [43]. ASPM-1 is required for oocyte meiotic spindle pole assembly and is essential for the meiotic spindles to align orthogonally and in close proximity to the overlying plasma membrane [43]. Another gene that may play a role in meiosis is *Bma-zyg-9* (Bm3504), which was down-regulated at the two highest IVM concentrations. In *C*. *elegans*, ZYG-9 acts to ensure correct microtubule assembly throughout the cell cycle of early embryos during mitosis, interphase and meiosis [44–46].

A major facilitator superfamily protein (Bm6076), down-regulated at 5 days at the two highest IVM concentrations, is highly expressed in the digestive tract of *B*. *malayi* [47]. In *C*. *elegans*, RNAi phenotypes associated with the ortholog of Bm6076 include “embryonic lethal”, “asymmetric cell division defective early embryo”, and “spindle pole pulling force variant”, indicating that this gene is probably important for embryo development.

Calcium signaling plays a crucial role in reproduction and fertilization and has been extensively studied in *C*. *elegans* [48]. During meiotic maturation of the oocyte, calcium signaling is linked to inositol 1,4,5-triphosphate receptor (ITR-1), N-methyl D-aspartate type glutamate receptor subunit (NMR-1) and UNC-43, which encodes a type II calcium/calmodulin-dependent protein kinase [49]. Phospholipase C catalyzes the hydrolysis of phosphatidylinositol 4,5-bisphosphate (PIP_2_) into diacylglycerol (DAG) and inositol (1,4,5) triphosphate (IP_3_) [50]. IP_3_ binds receptors on the endoplasmic reticulum to mobilize Ca^2+^ into the cytosol. We observed down-regulation of a phospholipase C homolog (Bm3716) and of 1-phosphatidylinositol-4,5-bisphosphate phosphodiesterase beta 4 (Bm1768). The latter is an ortholog of *egl-8* (egg-laying defective) and is predicted to have phosphatidylinositol phospholipase C activity and Ca^2+^ ion binding activity. Interestingly, EGL-8 also plays a role in regulating the release of acetylcholine from motor neurons, thus affecting locomotion, and is expressed in all neurons in *C*. *elegans* [51]. One of the most down-regulated DE genes we observed was inositol-1 (Bm11145), which is required for the biosynthesis of phosphatidylinositol [52]. Cumulatively, down-regulation of these genes suggests that Ca^2+^ signaling (via IP_3_) may be affected following exposure to IVM, with adverse effects on ovulation.

IVM selection on β-tubulin has been reported in *O*. *volvulus* and *H*. *contortus;* IVM selection changes the frequency of β-tubulin alleles [53]. Microtubules, which are assembled from heterodimers of α- and β-tubulin, drive chromosome motion in mitosis and meiosis [54], and so IVM selection for β-tubulin changes could have downstream effects on meiosis. A recent study in *H*. *contortu*s has also shown that IVM binds to and alter the tubulin polymerization equilibrium, which can lead to mitotic arrest [55].

A number of other genes that function in fertilization were dysregulated by IVM exposure. *Bma-emo-1* (Bm14080), which is involved in ovulation, intracellular protein transport and oocyte growth in *C*. *elegans*, was down-regulated. *C*. *elegans emo-1* mutants fail to ovulate after meiotic maturation and oocytes remain trapped in the gonad arm and become endomitotic [56]. Fanconi anemia complementation group D2 protein (Bm6522) was decreased following IVM treatment; deletion of the *C*. *elegans* ortholog results in egg-laying defects, precocious oogenesis, and partial defects in fertilization, and may play a role in double-stranded DNA break repair during embryogenesis [57].

### IVM exposure dysregulates the expression of genes involved in embryo development

IVM exposure downregulated the expression of several genes involved in RNA transport, with consequences on embryo development. Nuclear pore complexes (NPCs) are highly conserved proteinaceous structures embedded in the nuclear envelope that provide a connection between the nucleus and the cytoplasm [63]. Several genes (Bm6397, Bm9537, Bm5288) orthologous to NPC proteins in *C*. *elegans* (*npp-13*, *npp-14* and *npp-21* respectively) were down-regulated. Nucleoporins comprise NPCs and may function in transcriptional events, mRNA export, and genome organization [58]. In the nucleoplasm, dynamic nucleoporins may be functioning in the activation of developmental genes [58]. A subclass of nucleoporins plays a role in orienting the mitotic spindle in *C*. *elegans* embryos, and RNAi depletion of any of these nucleoporins leads to spindle orientation defects [64]. One of these nucleoporins is NPP-13. NPP-21, an ortholog of human TPR (translocated promoter region), is also involved in embryo development, locomotion and nematode larval development; RNAi knockdown results in embryonic lethal, larval arrest and locomotion variant phenotypes [59]. No obvious RNAi phenotypes have been reported for NPP-14.

Lastly, we observed down-regulation of Bm5608, which is orthologous to *mel-46* (maternal effect lethal). In *C*. *elegans*, this gene encodes a DEAD-box protein required in the germ line for proper oogenesis and zygotically for post-embryonic development [60].

### Pharyngeal pumping

Interestingly, we saw a significant down-regulation of *Bma-nep-1* (Bm7419), which has 45.8% identity to *nep-1* in *C*. *elegans*. Neprilysins are transmembrane zinc-metalloproteases that have roles in cell-to-cell signaling [61]. The expression pattern of *nep-1* in *C*. *elegans* is limited to pharyngeal cells and a single head neuron; it is an effector of locomotion and pharyngeal pumping [62]. The locomotion of *nep-1* knockouts is significantly impaired and RNAi knockdown results in a maternal sterile phenotype [62].

### Oxidative phosphorylation

We noted a significant enrichment of dysregulated genes involved in oxidative phosphorylation (100 nM, 48 h). Expression of ubiquinol-cytochrome C reductase (Bm6047), succinate dehydrogenase (Bm3783), ATP synthase epsilon chain (Bm3758), ATP synthase f chain (Bm5403), NADH dehydrogenase subunit 1 (Bm10539) and cytochrome b5-like heme/steroid binding domain containing protein (Bm2497) was down-regulated. In contrast, NADH dehydrogenase subunit 1 was up-regulated at 1 μM (48 h). The down-regulation of these genes may suggest that ATP synthesis is directly or indirectly inhibited by IVM. Abamectin (ABA), another ML in the avermectin family, caused concentration-dependent (5–25 μM) inhibition of the respiratory chain [63]. Reduced expression of ribosomal proteins, histones, and prosaposins [64] are a signature of oxidative stress in *C*. *elegans*, but the homologs of these genes were not DE or only marginally so in our study. In nematodes, ATP is obtained by glycolysis or oxidative phosphorylation, using a variety of substrates [65, 66]. Several anthelmintics have been proposed to target helminth oxidative phosphorylation enzymes [67].

### Glutamate-gated chloride channels

IVM binding to GluCls induces a slow onset, pseudo-irreversible glutamate-gated current [10, 68, 69]. Given the nature of the drug-receptor association, one can speculate on the necessity for a new cohort of GluCls to be expressed to overcome the effects of IVM. Little is known about the rate of receptor turn-over and synthesis in adult parasitic nematodes, but this scenario could contribute to the prolonged sterilization of adult filariae observed after a single dose of IVM. In this context, it might have been expected that this drug-receptor interaction would lead to increased expression of GluCl genes, which was not observed; expression of the putative receptor for IVM, *Bm-avr-14* (Bm1710 isoforms) [10], was not dysregulated following exposure to the drug. GluCl-encoding genes are abundantly expressed in *B*. *malayi* female reproductive tissue (among other body parts, such as the pharynx), suggesting that the sterilizing effects of IVM may occur at a very early embryonic stage [14]. In males, GluCl expression in reproductive tissues was less abundant, but was localized to some extent to somatic muscle and the *vas deferens*, likely controlling body movement as well as sperm cell development and release [14]. Using a high-level IVM-resistant *C*. *elegans* strain (the strain DA1316, which has null mutations in three GluCl subunits: *avr-14*; *avr-15*; *glc-1*) [70], no differential expression was noted in the genes encoding the GluCl subunits *avr-14* or *avr-15*, whereas a log fold-change of 0.78 (FDR = 0.06) in *glc-1* was observed following treatment with 1.1 μM IVM. Exposure to the drug impaired pharyngeal pumping to some extent in that strain. The authors concluded that IVM induced differential expression of several genes in response to food deprivation, which are likely unrelated to GluCl expression [70].

### Effects of *in vitro* culture on the *B*. *malayi* transcriptome

Twenty-nine cuticle collagen genes showed differential expression across both studies, of which 17 were significantly down-regulated following exposure to IVM. Previously, we examined the effect of *in vitro* cultivation, without anthelmintic, on the transcriptome of *B*. *malayi* and found 21 cuticle collagen genes to be significantly DE over time [20]. Thirteen of the DE collagen genes detected in the current work overlapped with dysregulated genes from the previous study (Bm11024, Bm11095, Bm11338, Bm1249, Bm2110, Bm2605, Bm2854, Bm4605, Bm7408, Bm7894, Bm8043, Bm8439 and Bm9021). Bm11024 and Bm11095 were significantly down-regulated in the *in vitro* cultivation study at 48 h in culture, yet in the present work, we found these genes to be up-regulated at 300 nM IVM for 5 days. Collagen gene dysregulation likely represents a non-specific marker of stress [20, 64]. Scanning electron microscopy has shown that IVM exposure leads to damage of the surface of *B*. *malayi* third-stage larvae, inducing the loss of regular cuticular annulations and causing morphological changes in the cuticular surface in the head, body, and tail regions [71]. Previously, we observed the pronounced up-regulation of a serpin precursor (Bm9380), and of a hypothetical protein (Bm337), attributed to *in vitro* cultivation [20]. In the current study, both genes were up-regulated, but to a much lower extent. This suggests that our untreated control corrects, to some extent, for differential gene expression solely due to life in liquid culture media.

### Comparison of the IVM transcriptional response in *C*. *elegans* to *B*. *malayi*

We compared the IVM transcriptional response in *C*. *elegans* to *B*. *malayi* to look for common GluCl-independent, IVM-dependent gene expression signatures. The transcriptional response to IVM in *C*. *elegans* using whole genome microarray led to the identification of genes involved in early food deprivation response (i.e., fat mobilization) [70]. Using the DA1316 mutant strain (null mutations in three GluCl subunits), which is resistant to the paralytic effects of IVM on the body wall, worms were exposed to 100 nM or 1.1 μM IVM for 4 h. This resulted in the up-regulation of 3 genes and down-regulation of 12 genes in the 100 nM IVM group, with 216 up-regulated and 153 down-regulated in the 1.1 μM group. Comparing our dataset with the *C*. *elegans* study showed that only 5 genes overlapped, of which 2 were significantly down-regulated in both studies: 6-pyruvoyl tetrahydropterin synthase (Bm1701) and cytochrome b5-like heme/steroid binding domain containing protein (Bm2497).

### Conclusion

We have shown that IVM exposure alters the expression of genes that are likely to function in the *B*. *malayi* female reproductive system. Through several biological pathways, genes involved in meiosis were particularly affected. These findings provide some insight into the mechanisms involved in IVM-induced reduction in mf output and impaired fertility, embryogenesis, and larval development and the transcriptomic dataset provides important experimental avenues to pursue the mechanism by which IVM sterilizes worms. This is also the first report of IVM exposure having an effect on oxidative phosphorylation at the transcript level, a metabolic pathway believed to be vital for nematodes.

## Supporting Information

S1 Table Pairwise comparisons of differentially expressed genes. The table shows the DE genes at each time point for comparisons between treated groups (100 nM, 300 nM, or 1 μM) and untreated groups at either 24 h, 48 h, 72 h, or 5 d IVM exposure for both studies.
(XLSX)

S2 Table Gene ontology enrichment tables for pairwise comparisons. Tables containing top enriched GO terms at 300 nM IVM exposure for 48 h vs untreated (A), 1 μM IVM exposure for 48 h vs untreated (B), 300 nM IVM exposure for 5 d vs untreated (C), and 1 μM IVM exposure for 5 d vs untreated (D).
(DOCX)

S3 Table Common dispersion and biological coefficients of variation (BCV) values for RNA-seq studies. Common dispersion and BCV values were calculated using the edgeR Bioconductor package (Version 3.12.0).
(DOCX)

S4 Table Enriched RNA interference phenotypes associated with down-regulated genes involved in reproduction. A hypergeometric test was performed to determine which phenotypes were significantly enriched when compared with their overall occurrence in the *C*. *elegans* RNAi dataset in Wormbase. P-values were corrected for multiple testing using Benjamini-Hochberg method (1995) with a level of significance of q<0.05.
(XLSX)

S1 Fig **(a-g) Biological coefficient of variation (BCV) vs read abundance (counts per million) for different pairwise comparisons.** Tagwise BCV values are plotted against the average log CPM values for each pairwise comparison performed in both studies.
(DOCX)
