# Supplementary material for: The Effects of Ivermectin on Brugia malayi Females In Vitro: A Transcriptomic Approach
Source: PLoS Negl Trop Dis. 2016 Aug 16;10(8):e0004929. doi: 10.1371/journal.pntd.0004929 (PMC4986938; doi:10.1371/journal.pntd.0004929)
Supplement: S2 Table — Tables containing top enriched GO terms at 300 nM IVM exposure for 48 h vs untreated (A), 1 μM IVM exposure for 48 h vs untreated (B), 300 nM IVM exposure for 5 d vs untreated (C), and 1 μM IVM exposure for 5 d vs untreated (D). (DOCX) [file pntd.0004929.s002.docx]

**S2a Table. Gene Ontology enrichment analysis of top 15 biological processes at 300 nM IVM exposure for 48 hours.**

| **Gene Ontology Term*** | **GO Identity** | **Observed** | **Reference** | **Ratio of Enrichment** | **FDR value**** |
| --- | --- | --- | --- | --- | --- |
| establishment of meiotic spindle orientation | GO:0051296 | 2 | 5 | 56.32 | 0.0345 |
| centrosome and pronuclear rotation | GO:0035047 | 2 | 6 | 46.93 | 0.0345 |
| regulation of tube size | GO:0035150 | 2 | 7 | 40.23 | 0.0345 |
| meiotic spindle organization | GO:0000212 | 2 | 8 | 35.2 | 0.0356 |
| establishment of meiotic spindle localization | GO:0051295 | 2 | 9 | 31.29 | 0.0356 |
| epithelial cell development | GO:0002064 | 2 | 9 | 31.29 | 0.0356 |
| epithelial cell differentiation | GO:0030855 | 2 | 10 | 28.16 | 0.0356 |
| regulation of ARF protein signal transduction | GO:0032012 | 2 | 11 | 25.6 | 0.0356 |
| ARF protein signal transduction | GO:0032011 | 2 | 11 | 25.6 | 0.0356 |
| energy reserve metabolic process | GO:0006112 | 2 | 12 | 23.46 | 0.0356 |
| glucan metabolic process | GO:0044042 | 2 | 12 | 23.46 | 0.0356 |
| cellular glucan metabolic process | GO:0006073 | 2 | 12 | 23.46 | 0.0356 |
| glycogen metabolic process | GO:0005977 | 2 | 12 | 23.46 | 0.0356 |
| establishment of spindle orientation | GO:0051294 | 3 | 34 | 12.42 | 0.0356 |
| locomotion | GO:0040011 | 22 | 1665 | 11.83 | 0.0356 |

*Analysis performed using Web-Based Gene Set Analysis Toolkit (WebGestalt). **Statistical method: Hypergeometric; p value adjusted by multiple test adjustment.

**S2b Table. Gene Ontology enrichment analysis of top 15 biological processes at 1 µM IVM exposure for 48 hours.**

| **Gene Ontology Term*** | **GO Identity** | **Observed** | **Reference** | **Ratio of Enrichment** | **FDR value*** |
| --- | --- | --- | --- | --- | --- |
| translation | GO:0006412 | 11 | 324 | 8.3 | 8.87E-06 |
| cellular macromolecule biosynthetic process | GO:0034645 | 14 | 1183 | 2.89 | 0.0073 |
| macromolecule biosynthetic process | GO:0009059 | 14 | 1186 | 2.89 | 0.0073 |
| gene expression | GO:0010467 | 15 | 1384 | 2.65 | 0.0109 |
| cellular biosynthetic process | GO:0044249 | 14 | 1457 | 2.35 | 0.0511 |
| organic substance biosynthetic process | GO:1901576 | 14 | 1478 | 2.32 | 0.0511 |

*Analysis performed using Web-Based Gene Set Analysis Toolkit (WebGestalt). **Statistical method: Hypergeometric; p value adjusted by multiple test adjustment.

**S2c Table. Gene Ontology enrichment analysis of top molecular processes at 300 nM IVM exposure for 5 days.**

| **Gene Ontology Term*** | **GO Identity** | **Observed** | **Reference** | **Ratio of Enrichment** | **FDR value*** |
| --- | --- | --- | --- | --- | --- |
| fatty-acyl-CoA binding | GO:0000062 | 2 | 10 | 70.3 | 0.0057 |
| structural constituent of cuticle | GO:0042302 | 6 | 161 | 13.1 | 0.0002 |
| structural molecule activity | GO:0005198 | 6 | 456 | 4.62 | 0.0165 |

*Analysis performed using Web-Based Gene Set Analysis Toolkit (WebGestalt). **Statistical method: Hypergeometric; p value adjusted by multiple test adjustment.

**S2d Table. Gene Ontology enrichment analysis of top 15 biological processes at 1 µM IVM exposure for 5 days.**

| **Gene Ontology Term*** | **GO Identity** | **Observed** | **Reference** | **Ratio of Enrichment** | **FDR value**** |
| --- | --- | --- | --- | --- | --- |
| double-strand break repair via nonhomologous end joining | GO:0006303 | 2 | 4 | 32.72 | 0.0228 |
| cytokinesis, completion of separation | GO:0007109 | 2 | 4 | 32.72 | 0.0228 |
| cytokinetic cell separation | GO:0000920 | 2 | 5 | 26.17 | 0.0305 |
| non-recombinational repair | GO:0000726 | 2 | 5 | 26.17 | 0.0305 |
| cytokinetic process | GO:0032506 | 3 | 8 | 24.54 | 0.0059 |
| nuclear-transcribed mRNA catabolic process, nonsense-mediated decay | GO:0000184 | 3 | 9 | 21.81 | 0.008 |
| intra-Golgi vesicle-mediated transport | GO:0006891 | 2 | 6 | 21.81 | 0.0367 |
| double-strand break repair | GO:0006302 | 3 | 11 | 17.85 | 0.0126 |
| polar body extrusion after meiotic divisions | GO:0040038 | 3 | 12 | 16.36 | 0.016 |
| regulation of DNA metabolic process | GO:0051052 | 4 | 17 | 15.4 | 0.005 |
| cytokinesis after meiosis | GO:0033206 | 3 | 13 | 15.1 | 0.0197 |
| female meiosis | GO:0007143 | 3 | 15 | 13.09 | 0.0228 |
| nuclear-transcribed mRNA catabolic process | GO:0000956 | 3 | 17 | 11.55 | 0.028 |
| organelle organization | GO:0006996 | 21 | 687 | 10.5 | 0.0259 |
| collagen and cuticulin-based cuticle development | GO:0040002 | 9 | 107 | 5.5 | 0.0032 |

*Analysis performed using Web-Based Gene Set Analysis Toolkit (WebGestalt). **Statistical method: Hypergeometric; p value adjusted by multiple test adjustment.
