# Supplementary material for: The Effects of Ivermectin on Brugia malayi Females In Vitro: A Transcriptomic Approach
Source: PLoS Negl Trop Dis. 2016 Aug 16;10(8):e0004929. doi: 10.1371/journal.pntd.0004929 (PMC4986938; doi:10.1371/journal.pntd.0004929)
Supplement: S3 Table — Common dispersion and BCV values were calculated using the edgeR Bioconductor package (Version 3.12.0). (DOCX) [file pntd.0004929.s003.docx]

S3 Table. Common Dispersion Values and Biological Coefficients of Variation (BCV) for RNA-seq Studies

|  | Pairwise Comparison | Common Dispersion | BCV |
| --- | --- | --- | --- |
| First Study | IVMT2 vs CtrlT2 | 0.02846000 | 0.1687000 |
|  | IVMT3 vs CtrlT3 | 0.04423981 | 0.2103326 |
|  | IVMT4 vs CtrlT4 | 0.07267216 | 0.2695777 |
| Second Study | IVM1T2 vs CtrlT2 | 0.12232850 | 0.3497549 |
|  | IVM2T2 vs CtrlT2 | 0.09236958 | 0.3039236 |
|  | IVM1T3 vs CtrlT3 | 0.02304176 | 0.1517951 |
|  | IVM2T3 vs CtrlT3 | 0.03223106 | 0.1795301 |
