# Supplementary material for: The Effects of Ivermectin on Brugia malayi Females In Vitro: A Transcriptomic Approach
Source: PLoS Negl Trop Dis. 2016 Aug 16;10(8):e0004929. doi: 10.1371/journal.pntd.0004929 (PMC4986938; doi:10.1371/journal.pntd.0004929)
Supplement: S1 Fig — (a-g) Biological coefficient of variation (BCV) vs read abundance (counts per million) for different pairwise comparisons. Tagwise BCV values are plotted against the average log CPM values for each pairwise comparison performed in both studies. (DOCX) [file pntd.0004929.s005.docx]

**S1 (a-g) Figures. Biological Coefficient of Variation (BCV) vs Read Abundance (counts per million) for different pairwise comparisons**


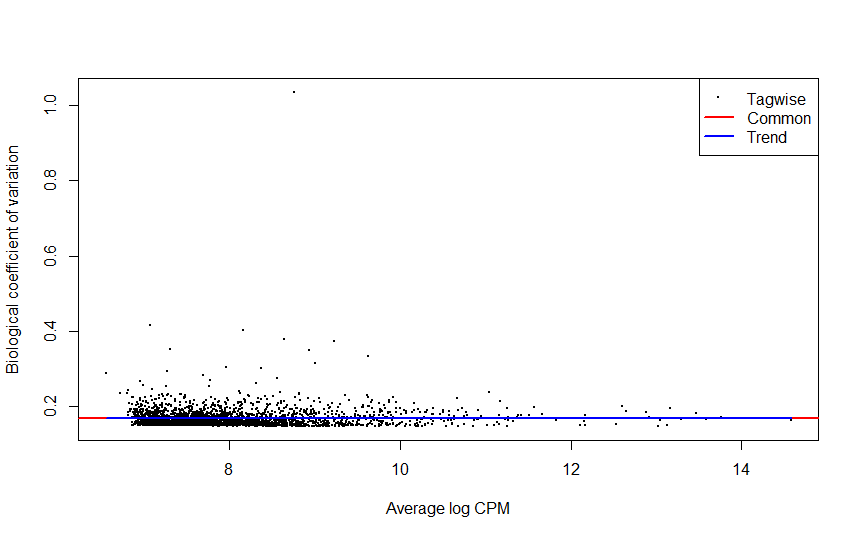


**S1a Fig. BCV Plot for IVM T2 vs Ctrl T2.** Tagwise BCV values are plotted against the average log CPM values. The red line shows the common BCV towards which the transcript-wise estimates (black dots) are 'squeezed' by the edgeR algorithm.


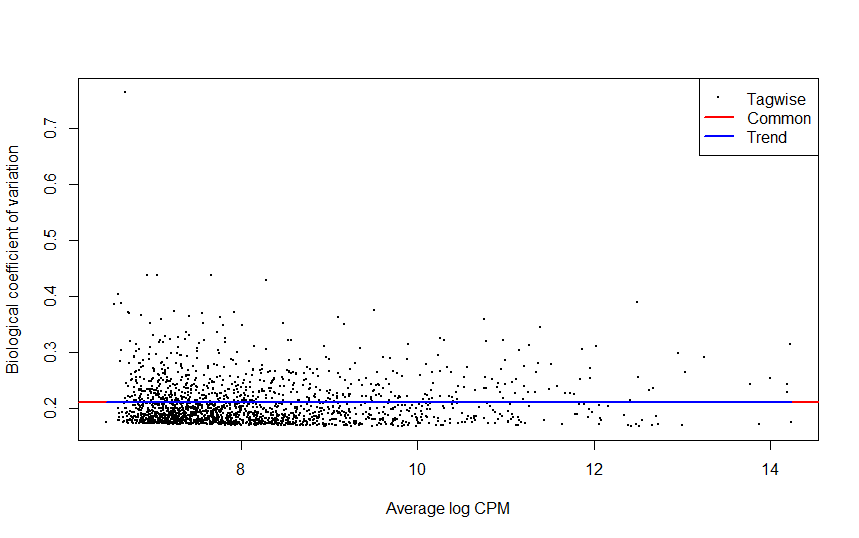


**S1b Fig. BCV Plot for IVM T3 vs Ctrl T3**. Tagwise BCV values are plotted against the average log CPM values. The red line shows the common BCV towards which the transcript-wise estimates (black dots) are 'squeezed' by the edgeR algorithm.


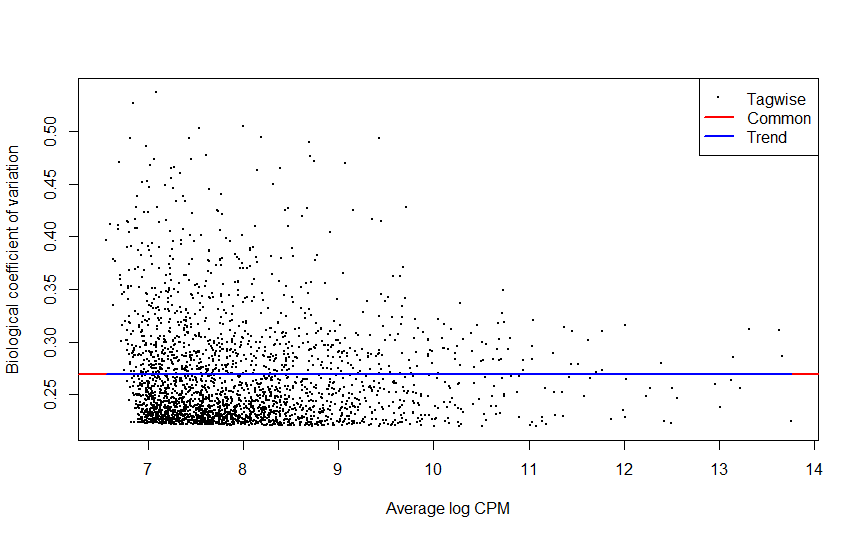


**S1c Fig. BCV Plot for IVM T4 vs Ctrl T4**. Tagwise BCV values are plotted against the average log CPM values. The red line shows the common BCV towards which the transcript-wise estimates (black dots) are 'squeezed' by the edgeR algorithm.


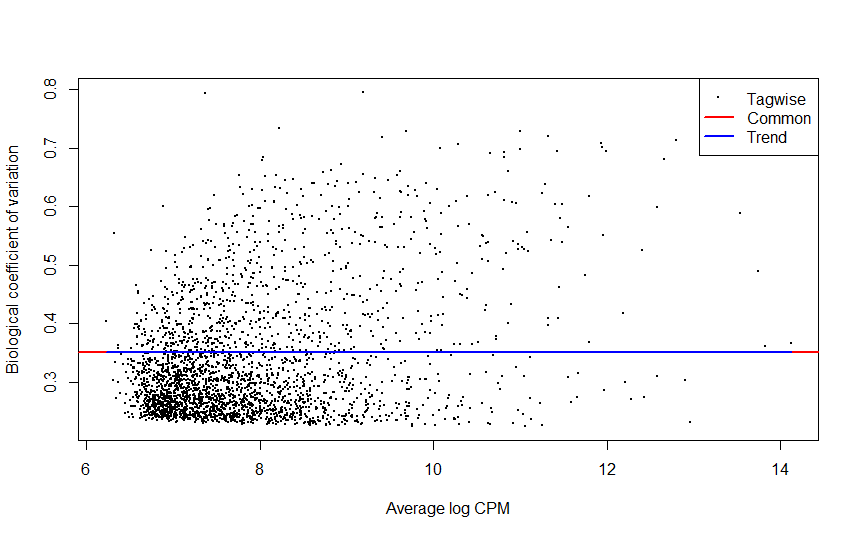


**S1d Fig. BCV Plot for IVM1T2 vs Ctrl T2.** Tagwise BCV values are plotted against the average log CPM values. The red line shows the common BCV towards which the transcript-wise estimates (black dots) are 'squeezed' by the edgeR algorithm.


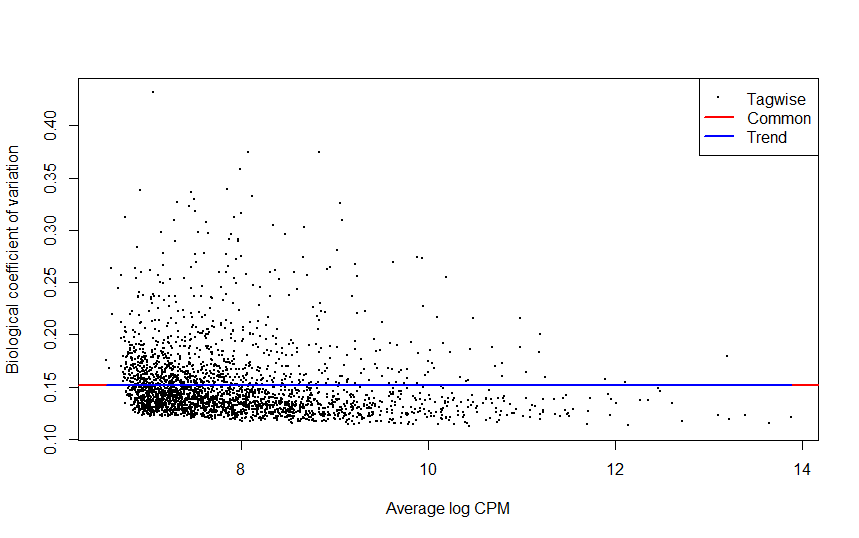


**S1e Fig. BCV Plot for IVM2 T2 vs Ctrl T2.** Tagwise BCV values are plotted against the average log CPM values. The red line shows the common BCV towards which the transcript-wise estimates (black dots) are 'squeezed' by the edgeR algorithm.


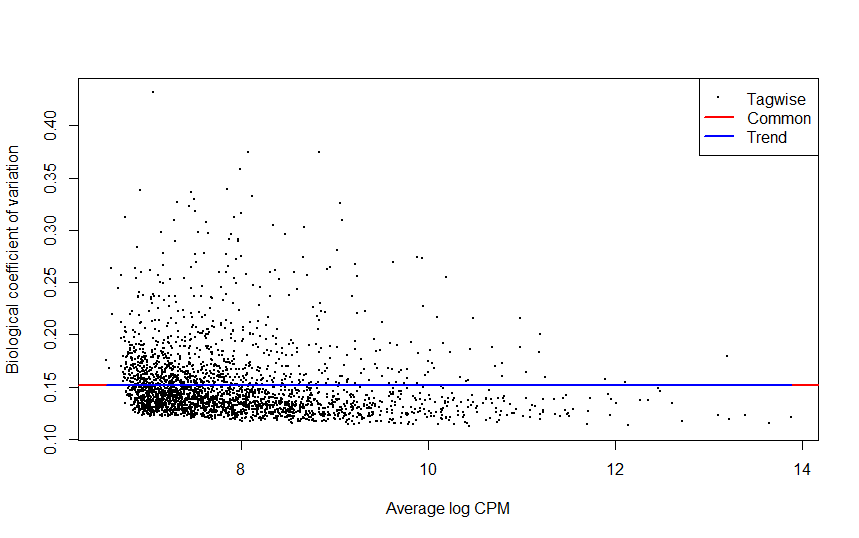


**S1f Fig. BCV Plot for IVM1 T3 vs Ctrl T3.** Tagwise BCV values are plotted against the average log CPM values. The red line shows the common BCV towards which the transcript-wise estimates (black dots) are 'squeezed' by the edgeR algorithm.


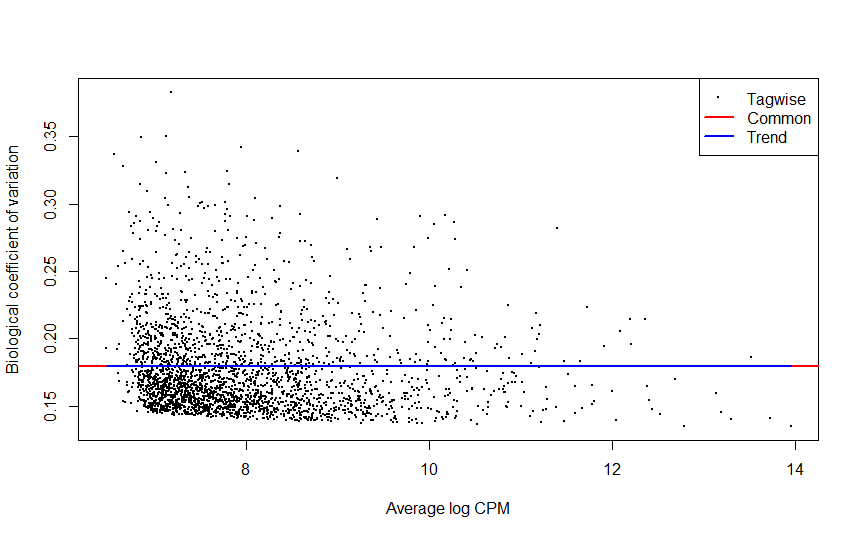


**S1g Fig. BCV Plot for IVM2 T3 vs Ctrl T3.** Tagwise BCV values are plotted against the average log CPM values. The red line shows the common BCV towards which the transcript-wise estimates (black dots) are 'squeezed' by the edgeR algorithm.
